# Ocean conditions drive interannual variability in juvenile albacore tuna (*Thunnus alalunga*) muscle energy content in the California Current System

**DOI:** 10.1371/journal.pone.0331436

**Published:** 2025-09-11

**Authors:** Catherine F. Nickels, Barbara A. Muhling, Elan J. Portner, Owyn E. Snodgrass, Heidi Dewar

**Affiliations:** 1 University of California, Santa Cruz, Institute of Marine Sciences’ Fisheries Collaborative Program, Santa Cruz, California, United States of America; 2 Fisheries Resources Division, Southwest Fisheries Science Center, National Marine Fisheries Service, National Oceanic and Atmospheric Administration, California, United States of America; 3 Scripps Institution of Oceanography, University of California San Diego, San Diego, California, United States of America; Hawaii Pacific University, UNITED STATES OF AMERICA

## Abstract

Juvenile albacore tuna (*Thunnus alalunga*) undertake long migrations across the North Pacific that they fuel by feeding in the California Current System (CCS) during the summer. The CCS is a highly dynamic system, which can lead to differences in foraging success that may be indicated by differences in body condition. Assessed through stomach content analysis, albacore diet composition by mean proportional weight showed high interannual variability including some years characterized by large increases in consumption of anchovy (2011 = 81%, 2017, 2022) and sardine (*Sardinops sagax*, 2022). We used a generalized additive model (GAM) to investigate the impact of albacore length, month of collection, diet composition, and environmental conditions on albacore muscle energy content. Two proxies for muscle energy content, the carbon to nitrogen ratio (C:N) and ash free dry weight (AFDW), were positively but weakly correlated. Albacore C:N increased with month, body size, and upwelling in the first half of the year, and decreased with increasing sea surface temperature. The mean energy density of prey and chlorophyll-*a* concentration were not important predictors of muscle energy content, indicating that albacore may be resilient to changes in prey composition and productivity. Sea surface temperature was the most important predictor of muscle energy content, which suggests that marine heat waves and projected future warming in the CCS may have detrimental effects on albacore body condition or the value of the CCS as a foraging habitat.

## Introduction

In mobile animals, energetic demand can drive migration behaviors [1]. The success of long-distance migrations to access food can be impacted by variability in the prey fields on foraging grounds, as well as climate driven shifts in phenology [2]. Tracking the success of foraging migrations can help identify vulnerabilities to climate change but can be difficult to monitor *in situ*. Quantifying body condition through analysis of predator tissues can be used as a proxy for foraging success and show how body condition may vary on different scales of space and time.

Energy density (ED) is a robust metric of a species body condition and a key trait of prey directly relevant to predator energetics [3]. Among common pelagic prey species, fish generally have higher ED than squids and crustaceans [4]. A diet rich in fish prey may therefore result in proportionally higher energy intake for pelagic predators, and healthier body condition. Other factors including size, spatial distribution, and degree of aggregation of prey species may also impact predator energy intake [5,6]. These factors are often also under the influence of environmental conditions, such as temperature, productivity, or the presence and magnitude of mesoscale oceanographic features [7,8].

The California Current System (CCS) is a productive yet highly variable eastern boundary current that is a key foraging ground for a broad range of highly migratory species [9,10]. In addition to seasonal variability, dramatic shifts in the oceanographic conditions and potential prey fields can occur over relatively short time scales. For example, a marine heat wave in 2014–2016 led to an anomalous zooplankton community structure characterized by decreased densities of crustaceans and increased densities of gelatinous species [11]. Variability in prey communities has been shown to impact the mortality and body condition of marine predators in the CCS including salmon (*Oncorhynchus* spp., [12]), California sea lions (*Zalophus californianus*, [13]), and sea birds [14].

Juvenile albacore tuna (*Thunnus alalunga*, hereafter “albacore”) migrate to the CCS as 1–3-year-olds from spawning grounds in the western and central Pacific. Before returning to their spawning grounds as adults, they move seasonally between the northern CCS in summer, and the offshore North Pacific in winter [15,16]. Albacore are opportunistic predators whose diets can vary dramatically over short temporal and spatial scales in association with environmental drivers [17]. However, it is unclear whether these differences in diet have predictable consequences for albacore body condition and thus value as targets of a fishery priced per pound. Albacore are targeted by a summer troll fishery off Oregon and Washington, which was the third most important fishery by revenue to these states during the 2010’s [18]. Their availability to the fishery is dependent on their horizontal and vertical distribution, which is influenced by the abundance, distribution, and identity of prey [16]. For example, foraging on mesopelagic species deeper in the water column may translate to less availability in surface waters where the troll fleet operates.

If feeding is successful albacore gain fat in their muscle tissues over the course of the season [19,20], which fuels both continued summer foraging and seasonal migrations to distant winter foraging grounds. Fat content in albacore muscle can vary substantially both within [19] and between years [20]. The usable energy stored in tissues can be approximated as the amount of fat relative to less energetically valuable components (water, protein, ash, carbohydrates) or the total ED of all muscle tissue components together. For example, the ratio between carbon and nitrogen (C:N) can be used as a proxy of fat in tissues [21], while the ash free dry weight (AFDW) can be used as a proxy of energy density [22].

In this study, our objectives were to examine the predictability and interannual variability in albacore muscle energy content in the CCS. We compared two metrics of muscle energy content (C:N and AFDW), then used multivariate modelling to quantify the influence of albacore size, month of collection, food intake (mean prey ED), and environmental variability on muscle energy content.

## Methods

### Albacore collection and processing

Albacore stomachs were collected offshore of Northern California, Oregon, and Washington from 2009 to 2022 between June and October (91% July – September) through partnerships with recreational and commercial fishers. Samples of white muscle tissue from the dorsal musculature as close to the head as possible (2.5–7.5 cm behind the head) were collected from the same fish from 2012 to 2022. No samples were collected in 2016. Recreational fishers used trolling artificial lures, and commercial fishers used troll or pole-and-line gear with anchovy *(Engraulis mordax*) as live bait. Fork length (FL, cm) was measured from the tip of the lower jaw to the outside edge of the fork in the caudal fin. Albacore weights (W, kg) were directly reported in 2021 and 2022 (85 total), which were used to establish an empirically derived relationship between FL and W (W = 0.0000211 * FL^2.986^, *R^2^* = 0.98). Reported weight was divided by the expected weight determined from the equation to calculate relative condition [23–25]. The relationship was also applied to calculate W from FL for the remaining measured albacore. Length measurement (14% missing), weight measurement (86% missing), and month of collection (17% missing) were not available for all individuals due to the limitations of sampling within existing supply chains that must balance other priorities. Most albacore were selected for sampling at the processing stage, and therefore specific capture locations were not available for the majority of fish. Stomachs and white muscle tissue were sampled after landing and either frozen before transport or kept on ice and frozen on arrival at the lab for later processing (see details below).

### Albacore muscle tissue energy content

Approximately 2-3g of muscle tissue per individual was freeze-dried for a minimum of 24 hours and stored in a closed vial until analysis. Dry tissue was subsampled (1 ± 0.2 mg) and sent to the University of California Davis Stable Isotope Facility for measurement of C and N content using an elemental analyzer (Sercon Europa ANCA-GSL, Sercon Ltd., Cheshire, UK), from which we calculated C:N mass ratios. Samples of tissue from 20 albacore were analyzed per year from 2012–2022, except for 2018 where only 17 were available. Two samples (1% of the total analyzed) with C:N deemed to be unrealistic for fish white muscle tissue (C:N > 14 [26], 1 from 2012, 1 from 2014) were excluded from further analysis. After at least 24 hours in a desiccator, a separate subsample of approximately 1 gram of freeze-dried tissue was placed in a 600°C muffle furnace for 20 hours. The remaining ash was weighed and that value was subtracted from the initial dry weight to obtain ash free dry weight (AFDW [27]). Full combustion was determined by ash color. Samples that were not completely combusted were returned to the furnace with a small amount of distilled water for cycles of 20 hours until only ash remained (maximum number of cycles = 5 [27]).

We report AFDW as a percentage of dry weight (DW). Albacore white muscle tissue was stored frozen at −20°C for an extended period of time (up to 9 years), making wet weights unreliable [28]. Only 2014, 2021, and 2022 had sufficient tissue available for AFDW determination (*n* = 26, 29, 28, respectively). In instances where C:N and AFDW were available from the same fish we compared metrics using linear regression by year (2014: *n* = 9, 2021: 15, 2022: 20). We investigated the significance of year (as a categorical variable) and C:N in predicting AFDW through one way analysis of covariance (ANCOVA). C:N and AFDW were also compared to relative condition using linear regression when weight was measured in 2021 and 2022. As C:N data were available for more years than AFDW (10 vs 3 years, respectively), all subsequent analyses use C:N, a fat proxy, as an indicator of albacore muscle energy content.

### Diet analysis

The contents of thawed stomachs were rinsed over 0.5 mm brass mesh sieves and stored in 70% ethanol until prey were identified and enumerated using a dissecting microscope. Prey were identified to the lowest possible taxonomic level [29–35], primarily from hard parts due to the high degree of digestion. Fresh anchovy, which were larger and less digested than other prey, were presumed to be bait and excluded from prey counts.

Up to 5 individuals of each prey species per stomach were measured to the nearest 0.1 mm. Fish were measured as standard length (SL) when found whole and vertebral column length when digested, which was converted to SL following Glaser et al. [36]. Cephalopod beaks were measured as lower rostral lengths for squids and upper hood lengths for octopuses, which were then converted into mantle lengths using published regressions (S1 Table). Crustaceans were measured for SL when whole [32]. Prey lengths were converted into weight following Portner et al. [37] using taxon-specific equations from the literature (S1 Table). 7% of all prey were measured. Unmeasured prey were assigned the mean weight measured from the same prey group within the same stomach, year, or the whole data set in a hierarchical manner based on available measurements.

For each stomach, the mean energy density of consumed prey (ED_mean_) was calculated as the ED of each prey type weighted by its proportional contribution to the total weight of all prey in that stomach. Proportional prey weight was calculated as the total estimated weight per prey group in a stomach divided by the total estimated weight of all prey in that same stomach. Estimated proportional weights were multiplied by the taxon-specific energy densities (kJ g^-1^ wet weight) from the literature (S1 Table) and summed to get ED_mean_. Percent predator body weight consumed (%W) was calculated as the weight of all prey divided by albacore weight for each predator. Stomachs with a calculated %W exceeding the highest measured %W of a fully distended stomach during processing (>4%W) were excluded from analysis as unrealistic. A total of 10 stomachs (1.87% of stomachs containing prey) exceeded this threshold and were excluded to reduce potential bias from estimation uncertainty. Data handling and statistics were done in R version 4.3.3 [38].

### Patterns of variability in Albacore muscle tissue energy content

To determine whether albacore gained muscle energy content as the feeding season progressed, we first plot C:N vs month by year for samples where month was recorded (*n* = 135). We then examine whether albacore muscle energy content was related to food ED_mean_ and/or environmental variability by quantifying the effects of potential drivers using a generalized additive model (GAM) in the *mgcv* package v1.9-1 in R [39]. GAMs are non-parametric additive regression models, which use smoothing functions and can accommodate non-linear relationships between the dependent and independent covariates [40]. The independence of all covariates in a GAM facilitates interpretation because the effect of each covariate is not influenced by the others. Month was included in the model to capture potential increases in muscle energy content with time spent feeding in the system. Albacore FL was also included to account for an increase in fat content with size [20]. Only muscle tissue samples from individual albacore with complete data for ED_mean_, month, and FL were included in analysis (*n* = 113).

Potential environmental drivers of variability in muscle energy content (indicated by C:N) included sea surface temperature (SST), surface chlorophyll-*a* (Chl-*a*, as a proxy for standing phytoplankton biomass), and the Cumulative Upwelling Transport Index (CUTI [41]). SST and Chl-*a* are important predictors of the distribution of both albacore and several important prey species [42] and may influence the spatial overlap of predator/prey habitats. CUTI was included as a predictor because upwelling intensity early in the year can impact the abundance and distribution of prey later in the year [43,44]. Monthly SST and Chl-*a* were accessed in R using the *rerddapXtracto* package [45] from the Environmental Research Division’s Data Access Program (ERDDAP). Datasets include the Multi-scale Ultra-high Resolution (MUR) SST Analysis fv04.1, Global, 0.01, 2002–present, Monthly [46], Chlorophyll-*a*, Aqua MODIS, NPP, L3SMI, Global, 4 km, Science Quality, 2003–present (Monthly Composite) for 2009–2021 and Chlorophyll-*a*, Aqua MODIS, NPP, L3SMI, Global, 4 km, R2022 NRT, 2003-present (Monthly Composite) for 2022 [47]. SST and Chl-*a* were spatially averaged over the United States Exclusive Economic Zone (EEZ; 200 nautical miles offshore) between 40.45°N and 50°N, and temporally averaged over the July-August-September core fishing season (SST_f_, Chl-*a*_f_,). The EEZ contained 88% of reported catch locations, with the remainder further offshore within the same latitudinal boundaries. CUTI values were obtained from the NOAA Environmental Research Data Services website (https://oceanview.pfeg.noaa.gov/products/upwelling/dnld). CUTI values represent averages between 41°N and 47°N during the first 6 months of the year of capture as a prey preconditioning index (CUTI_pre_).

Correlation coefficients between predictors were low (Pearson’s *r*, > – 0.4 and < 0.4), thus all predictors were included in the same model (S1 Fig). The GAM was fit using restricted maximum likelihood parameter estimation. The GAM used a Gamma distribution with a log link function. Smooth terms included predator fork length, month of capture, ED_mean_, SST_f_, Chl-*a*_f_, and CUTI_pre_. We used the double penalty approach of Marra & Wood [48] to penalize spurious predictors out of the GAM. Model diagnostics and residuals were checked for potential deviations from normality, homogeneity of variance, and other anomalies.

## Results

### Comparing metrics of predator muscle energy content

**Fig 1:**
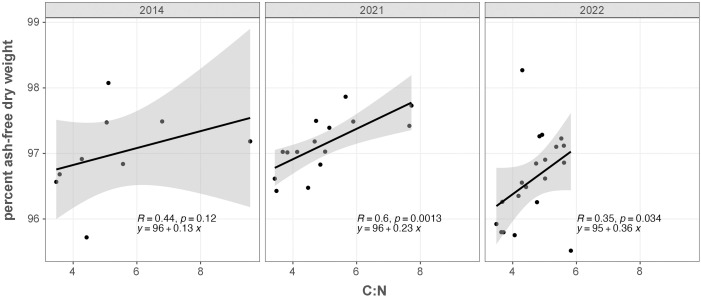
Comparison of C:N mass ratio and AFDW in years when both metrics were available. Each point represents an individual albacore. Black lines indicate linear regressions of AFDW vs C:N mass ratio by year, and grey shading represents 95% confidence intervals. 2014 *n* = 9, 2021 *n* = 15, 2022 *n* = 20.

C:N and AFDW (proxies for fat and energy content, respectively) were weakly but positively correlated, with the strength of the association variable by year (Fig 1). 2021 had the strongest relationship (*R*^*2*^ = 0.36, *p *< 0.01, *n* = 15), while 2022 was strongly positive but included two pronounced outliers (*R*^*2*^ = 0.12, *p* < 0.05, *n* = 20). 2014 had a weaker relationship than the other years (*R*^*2*^ = 0.19, *p *> 0.05, *n* = 9), likely due to the smaller sample size. When all years were analyzed together through ANCOVA, the categorical variable of year (*F*(2, 38)= 2.88, *p *> 0.05) and the interaction term between year and C:N (*F*(2, 38)= 0.65, *p *> 0.05) were not significant, indicating that the mean and slope did not differ between years. C:N still had a significant effect (*F*(1, 38)= 10.78, *p *< 0.01, linear regression all years pooled %AFDW = 96 + 0.23C:N, *R*^*2*^ = 0.14, *p *< 0.001, *n* = 44). AFDW did not vary substantially across the three years of available data (median values 2014 = 97 ± 0.92, 2021 = 97 ± 0.63, 2022 = 96.8 ± 0.66; Fig 1 and S2 Fig). C:N was also relatively stable for the three years where both data sets were available (median values 2014 = 4.56 ± 1.41, 2021 = 4.74 ± 1.24, 2022 = 4.75 ± 0.72; Fig 1 and S2 Fig). Relative condition was not significantly related to either C:N or AFDW (C:N: *R*^*2*^ < 0.01, *p *> 0.05, *n* = 39, AFDW: *R*^*2*^ < 0.001, *p *> 0.05, *n* = 57), and did not vary substantially across the two years of available data (median values 2021 = 0.998 ± 0.076, 2022 = 0.993 ± 0.053; S2 Fig). Henceforth we use C:N as an indicator of Albacore muscle energy content in analyses, due to a larger available sample size (C:N, *n* = 195; AFDW, *n* = 83, S2 Table).

### Annual patterns in diet metrics and environmental variables

**Fig 2:**
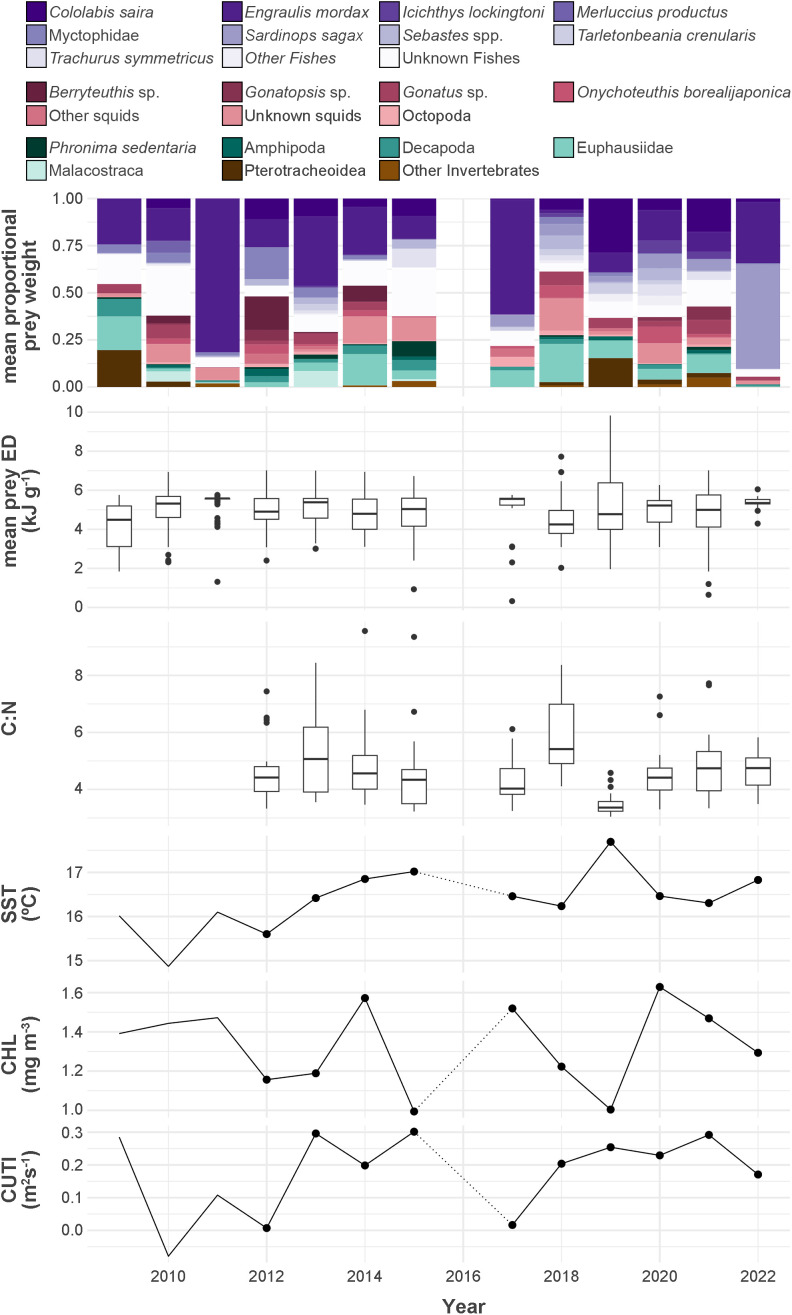
Time series. Time series of albacore diet composition as estimated mean proportional prey weight, prey ED_mean_, C:N mass ratio, SST_f_, Chl-*a*_f_, and CUTI_pre_. Points in the SST_f_, Chl-*a*_f_, and CUTI_pre_ plots indicate years included in the GAM.

Annual albacore diet composition, Prey ED_mean_, C:N, SST_f_, Chl-*a*_f_, and CUTI_pre_ are shown in Fig 2. A total of 612 albacore stomachs were processed across all years of the study, with 524 suitable for inclusion in diet analysis based on the following criteria: 1) contain prey and, 2) realistic estimate of %W (S3 Table). Albacore diet composition (mean proportional weight) showed high interannual variability including some years characterized by large increases in consumption of anchovy (2011 = 81%, 2017 = 61%, 2022 = 33%) and sardine (*Sardinops sagax*, 2022 = 55%). Years with smaller increases in the consumption of heteropods (Pterotracheoidea) were also apparent (2009 = 20%, 2019 = 15%). The relative contribution of cephalopods was also highly variable, exceeding 30% in 2012, 2014, and 2018. Diet variability was reflected by differences in the interquartile ranges of annual stomach ED_mean_s, with the lowest variability observed during years of strong anchovy or sardine consumption (2011, 2017, and 2022). During these years, ED_mean_ was consistently above 5 kJ g^-1^. Prey ED_mean_ for the entire time series was 4.9 kJ g^-1^ and did not fluctuate substantially between years.

C:N varied both within and between years across the time series. Annual means were highest in 2013 (5.3) and 2018 (5.88), and lowest in 2019 (3.49). 2019 also had the smallest range (3.05–4.58). SST_f_, Chl-*a*_f_, and CUTI_pre_ showed substantial interannual variability during our study period. 2019 was the warmest year included (17.69°C), with low Chl-*a*_f_ (1.00 mg m^-3^) and relatively high CUTI_pre_ (0.25 m^2^ s^-1^). 2012 was the coolest year with C:N data available (15.60°C), with the lowest CUTI_pre_ (0.01 m^2^ s^-1^) and moderately low Chl-*a*_f_ (1.56 mg m^-3^). The highest CUTI_pre_ (17.02 m^2^ s^-1^) was observed in 2015, when Chl-*a*_f_ was lowest (0.99 mg m^-3^) Chl-*a*_f_ reached a maximum peak (1.63 mg m^-3^) in 2020.

### Muscle energy content across the fishing season

**Fig 3:**
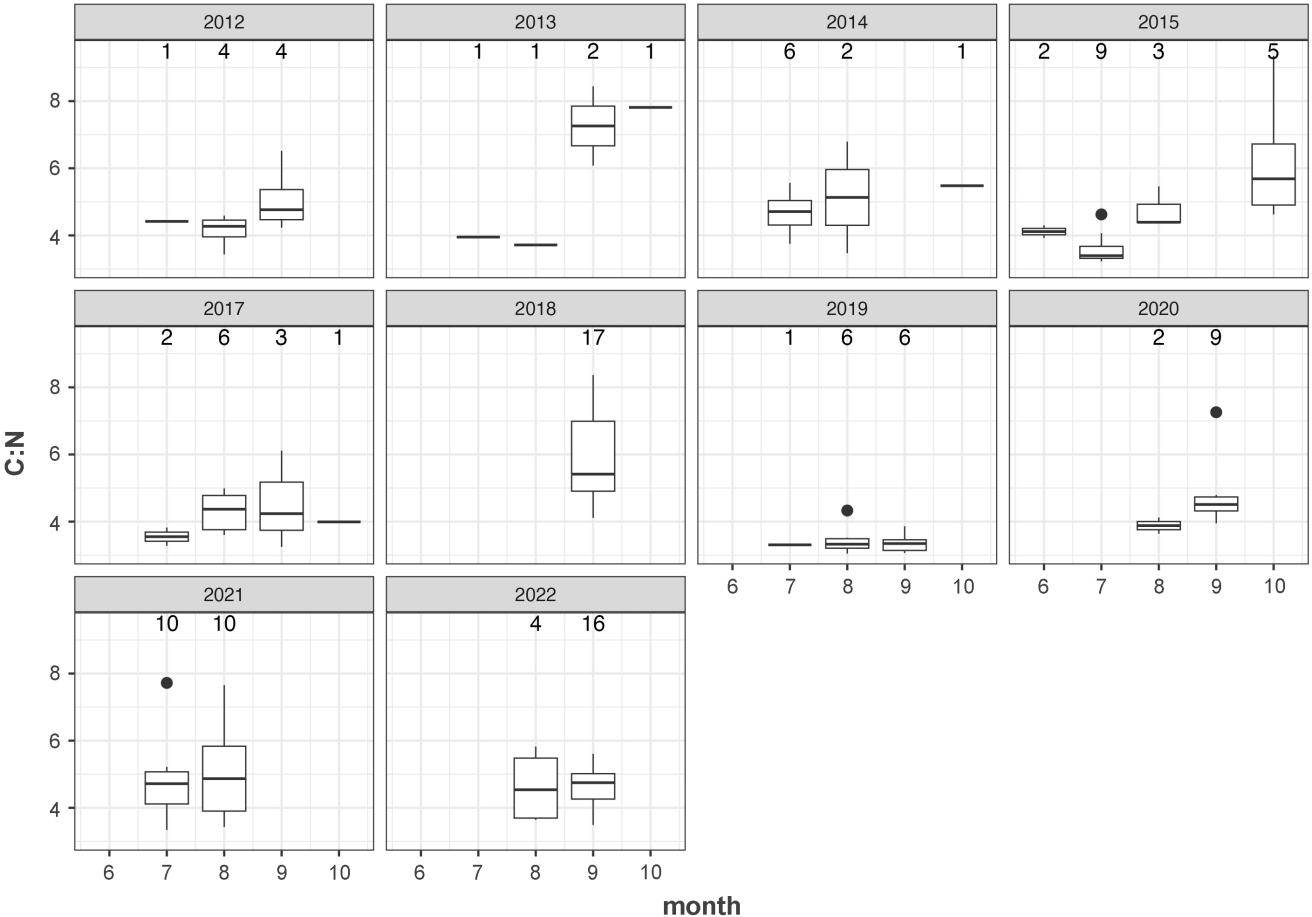
Boxplots of C:N mass ratio across months and years. Sample sizes are shown above each box.

Low sample size and inconsistent monthly coverage precluded quantitative analysis of the patterns in C:N by month within each year individually; however we note some qualitative trends here (Fig 3) and include month as part of the GAM below. Albacore appeared to gain fat (thus energy content, as indicated by higher C:N) throughout the summer fishing season. Median C:N across all years was 4.1 in June, 4.0 in July, 4.1 in August, 4.8 in September, and 5.6 in October. However, this pattern was not consistent within all individual years. In 2012–2015, 2020, and 2021, fish sampled later in the season had higher C:N ratios, whereas in 2017, 2019, and 2022 C:N did not increase with month.

### Predictors of muscle energy content

**Fig 4:**
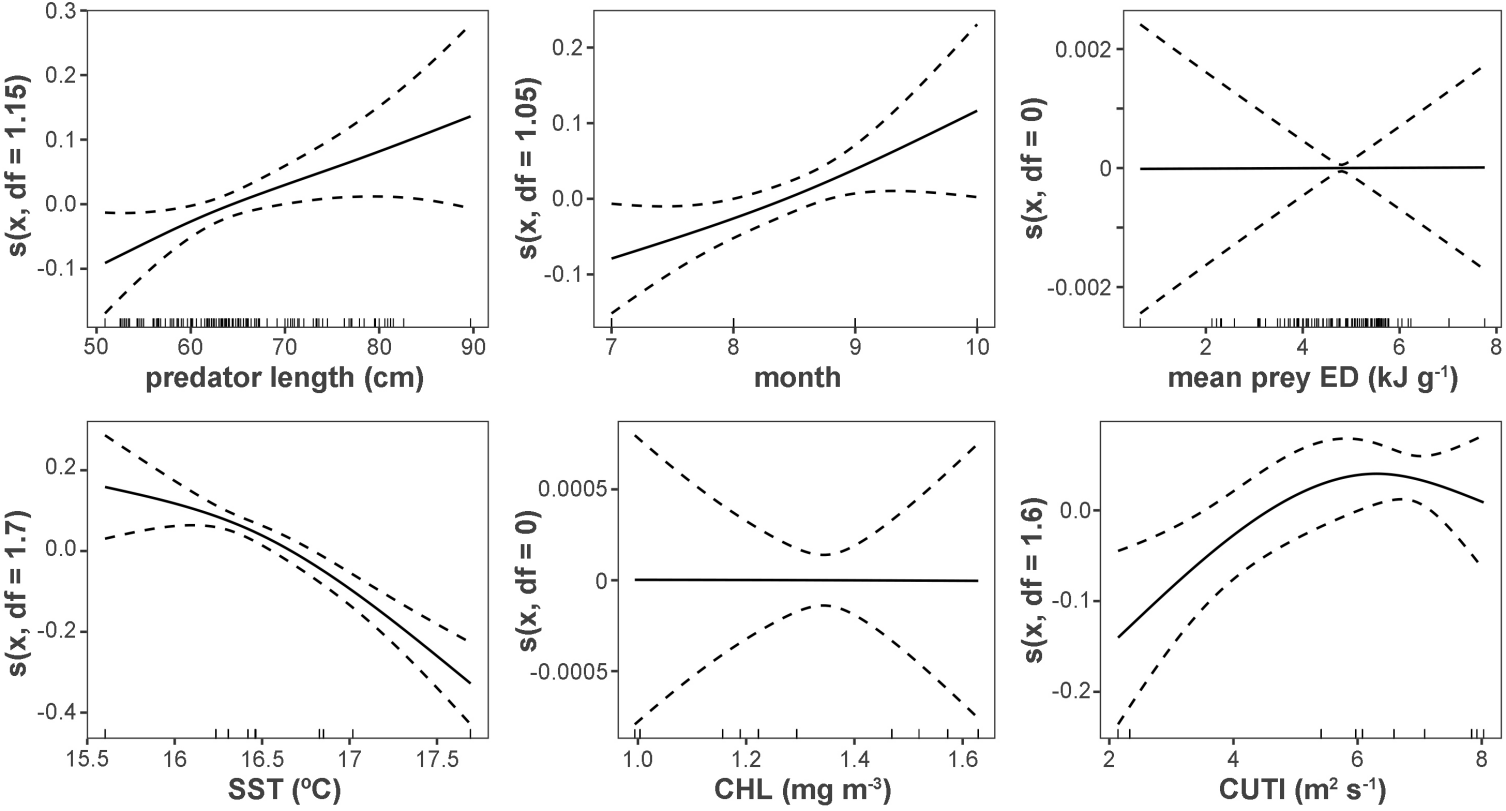
GAM partial effect plots. Model results of the partial effect of predator length, month, temperature, prey mean energy density, Chl-*a*, and CUTI on individual muscle tissue C:N of albacore. Deviance explained = 39.9%. Fitted lines and 95% confidence intervals (dashed lines) are shown; whiskers on the x-axis are observations for that covariate. *n* = 113.

Generalized additive modeling revealed the influence of environmental conditions on variation in albacore muscle tissue C:N, an indicator of energy content (Table 1, Fig 4; deviance explained = 39.9%). C:N was strongly affected by temperature, increasing as SST_f_ decreased. C:N also had positive relationships with predator fork length, month, and CUTI_pre_. ED_mean_ and Chl-*a*_f_ were not significant predictors of C:N, and were penalized out of the model.

**Table 1:** Summary of covariate contributions to GAM.

*C:N ~ s(predator fork length, k = 10) + s(month, k = 3) + s(ED*_*mean*_*, k = 10) + s(SST*_*f*_*, k = 3) + s(Chl-a*_*f*_*, k = 3) + s(CUTI*_*pre*_*, k = 3), n *= 113, *Adj R*^*2*^* = *0.32			
**Parametric coefficients**	**estimate**	***t*-value**	***p*-value**
Intercept***	1.54	78.62	<0.001
**Smooth terms**	**edf**	***F*-value**	***p*-value**
s(predator fork length)**	1.10	0.75	<0.01
s(month)**	1.05	2.95	<0.01
*s(ED*_*mean*_)	0.00	0.00	0.50
*s(SST*_*f*_ )***	1.70	21.00	<0.001
*s(Chl-a*_*f*_)	0.00	0.00	0.32
*s(CUTI*_*pre*_ )**	1.60	4.22	<0.01

Value estimate, *t*-value, and *p*-value given for the parametric coefficient intercept. Estimated degrees of freedom (edf), *F*-values, and *p*-values given for each smooth term. * after coefficient or term name reflects statistical significance based on *p*-value: ‘***’ denotes significance at p < 0.001, ‘**’ at p < 0.01, ‘*’ at p < 0.05.

## Discussion

Access to a long time series that included both stomach contents and muscle tissue samples allowed for the examination of patterns in body condition as indicated by muscle energy content. We investigated whether variability in predator size, month, diet, and environmental factors have a measurable impact on the muscle energy content of juvenile albacore. Albacore muscle tissue C:N increased with month, albacore size, and pre-season upwelling (CUTI_pre_) and decreased with increasing fishing season sea surface temperature (SST_f_). Neither the mean energy density of prey (ED_mean_) nor fishing season Chl-*a* (Chl-*a*_f_) were significant predictors of C:N. C:N and AFDW of albacore muscle tissue, proxies for tissue lipid content and energy density, respectively, were positively, though weakly, related.

### Mechanistic interpretation of relationships

Albacore fat content has been observed to increase throughout the foraging season in the California Current [19] and in the Bay of Biscay [20]. The increase over the course of the season indicates that the fish are feeding successfully and adding to their fat reserves for use as energy for migration, growth, and maturation. The positive relationship we observed between C:N and fish size is consistent with the observations of increasing fat content with fork length for albacore of similar size in the Bay of Biscay [20]. Albacore likely exhibit a decrease in growth rate with increasing length, thus as the fish become larger, they store more ingested energy as fat reserves instead of utilizing it for rapid growth [20].

The northeast Pacific Ocean has experienced longer and more frequent marine heatwaves over the past 15–20 years relative to the late 20th century [49]. Notable summer marine heatwaves have occurred in the CCS in most years since 2013 [50,51]. In recent years, marine heatwave conditions have peaked during summer, and subsided during fall [51], overlapping with peak fishing season for albacore. Temperature can impact tuna metabolic rate directly [52,53] or through indirect effects on prey availability or quality, including changes in spatial aggregation or distribution. Along with SST, upwelling can impact the spawning timing of important prey species including anchovy and sardine [54]. Upwelling early in the year may act as a prey preconditioning factor, influencing the sizes and nutritional value of prey available when the albacore arrive to feed [44]. During recent marine heatwaves, the trophic pathways supporting pelagic prey species in the northern CCS shifted towards gelatinous, less nutritious zooplankton [11]. If prey are in poor condition during warm events because of a shift in the food web [11,55], then predators may gain less energy during these times, even without any change in diet composition.

The local environmental variables included in the GAM are also related to larger, basin scale indices that could lead to interannual variation in the muscle energy content of albacore on arrival in the CCS through carryover effects of conditions during their winter offshore phase. Summer SSTs are more weakly connected to persistent, basin-scale patterns than winter SSTs, due to stronger stratification and lower thermal inertia during summer [56]. However, summer SST is impacted by El Niño-Southern Oscillation (ENSO) variability [57], as well as non-ENSO basin-scale circulation and regional winds [58]. The broad polygon used in this study captures both the extent of cooler, nearshore upwelled waters and the temperature of offshore oceanic waters, both of which are likely important to predator and prey distribution and overlap [58,59].

We did not find an impact of ED_mean_ of prey on predator muscle energy content, which could be the result of several non-exclusive explanations. While juvenile albacore generally select high energy prey [3], other factors such as size composition, spatial distribution, and schooling behavior can impact realized predator diet composition and net energy gain [5,6,60]. For example, larger forage fish may be more efficient for predatory tuna to capture and consume, the absence of which can result in unexpectedly poor predator body condition even when smaller members of prey species are abundant [6]. A shift in size composition would impact predator body condition without being reflected in dietary composition or ED_mean_. Juvenile albacore prey also tend to exhibit schooling behavior [3]. More densely packed prey facilitate more efficient feeding [60], which reduces the energy expended and time required to consume the predator’s daily ration. Lastly, the actual ED of consumed prey may have varied under the influence of environmental factors [11,55], which was masked by our use of static ED values from the literature for analysis.

### Caveats and limitations

While we examined both C:N and AFDW as indications of albacore muscle energy content, the measurements were only weakly positively related. Several factors could have contributed to the observed discrepancies. Both C:N and ADFW are proxies for fat or energy content, but are not direct measurements of either nutritional quality metric which may add noise to the relationship. We may have found discrepancies even if we were able measure lipid and energy density directly. While lipid is a major contributor to the caloric content of fish tissue, its actual caloric equivalent varies based on the composition of saturated and unsaturated fat components [61]. Thus, estimates of energy density inferred from proximate composition and singular caloric equivalents for each component can differ significantly from direct measurements using bomb calorimetry [62]. In addition, available muscle tissue was from the dorsal part of the head. Whole body or belly analysis may result in different conclusions due to differential body composition [63]. Even small differences in the collection of tissue by different processors could impact composition. We predicted C:N in our model due to the larger number of years sampled, and it may be a better metric when considering muscle only rather than whole fish. In whole fish, scales and bones contribute less to the total compared to muscle as body condition increases, which increases %AFDW. Whole body metrics are less practical in large fish like albacore than smaller fish like anchovy. We assume here that muscle tissue energy content reflects albacore health and total body condition, but that assumption has not been tested. Neither C:N nor %AFDW showed a significant relationship with the morphometric relative condition factor, but this analysis only included the two years where weights were directly reported (2021 and 2022) and spanned a limited range of values.

We did not find an impact of diet composition, summarized here as ED_mean_, on the muscle energy content of albacore. Total energy consumed over the course of a day may have been a more informative metric, however we determined that albacore stomach contents generally reflect less than a full day’s feeding. Compared by weight, estimates for tuna daily ration hover around 2% albacore body weight [64–66]. 92% of albacore with prey contained less than that benchmark, while 40% had less than 0.2% albacore body weight. In addition, the average total kJ per albacore stomach was estimated to be 235.3 kJ, which is much less than the 3766 kJ that Whitlock et al. [53] estimated that similarly sized Pacific bluefin tuna (*Thunnus orientalis*) consumed per day in the CCS. By both approaches, the average values are consistently less than the benchmarks expected from the literature, which indicates that our opportunistically collected samples underestimate the total daily consumption of juvenile albacore in the region. While it is theoretically possible to extrapolate a full day’s consumption from stomach contents consumed over a limited part of the day, the required calculations were not possible in this case. Albacore primarily feed during the day [16] and we have no record of the time each fish was collected. We therefore cannot differentiate between a fish collected early in the morning, before it has engaged in most of its feeding activity for the day, and a fish collected later in the day that encountered few potential prey. In addition, tunas can digest their prey rapidly [67], leading us to use reconstructions rather than direct measurements of prey weights and potentially missing contributions from soft bodied organisms that lack hard components.

The different timescales over which stomach contents and muscle energy content integrate may also influence the connection between the two metrics. In albacore, stomach contents reflect energy consumed over part of a day while muscle content in tunas has been found to integrate on the timescale of months [68]. The time lag can lead to a mismatch between stomach contents and muscle energy content in highly mobile predators during transitions between locations and/or prey [69]. Albacore have also been shown to be highly opportunistic, with variation in stomach contents on small scales of time and space [17]. While stomach contents reflect a single meal, individual albacore likely consume more varied prey over the period of time represented by muscle tissue.

Our samples were collected opportunistically through partnerships with commercial fishers, which provided a long time series but introduced some analytical challenges. A designed survey with monthly sampling could resolve some of the uncertainties by providing a more complete accounting of the impact of month on fish muscle energy content compared to other factors varying by year. Direct measurements of weight across a wider range of environmental variability would allow for a more robust comparison of morphometric and muscle energy content indicators of body condition. More detailed records may be able to clarify the impacts of time of day on stomach fullness.

### Broader implications

The ability of juvenile albacore to meet their energetic needs is more directly related to environmental factors than to the composition of their diets, which reflects a resilience to shifts in prey composition. However, their muscle energy content was lowest in the warmest year sampled (2019), suggesting that warmer conditions in the CCS may be detrimental for albacore in the region.

The CCS is projected to continue to warm substantially over the coming decades [70]. Coastal upwelling is projected to strengthen in the northern CCS, but there is uncertainty about the impacts of this increase on nutrient concentrations and primary productivity. Different climate models also disagree on changes in upwelling season phytoplankton biomass [71]. Given that albacore have diverse diets [17,72], it is difficult to anticipate how prey availability and energetic landscapes might change in the future. Albacore forage in the CCS as juveniles to gain energy and improve body condition before returning to the subtropical western North Pacific to spawn [16]. Years such as 2019, where albacore did not appear to improve in muscle energy content across the summer, may have implications for longer-term migratory patterns if this becomes more the norm in future. The mean C:N in 2019 of 3.49 approached the value of pure collagen protein (3–3.3, [73]), indicating that the fish were in poor condition. Our finding of low C:N in 2019 was in alignment with observations by fishers, who reported that albacore were thinner than usual that year (Rick Goche, Aquatic Resources Inc., pers. comm.).

Annual albacore landings in Oregon and Washington are highly variable [18]. Years of high or low landings are often not related to stock-wide recruitment or biomass, so variability in fish movements is assumed to be important. Frawley et al. [18] show that the fishery has moved substantially over time to follow concentrations of fish. These shifts could be driven by factors such as prey availability, but clear evidence has not yet been identified [17]. The present study suggests that environmental variability may influence albacore availability to the fishery if albacore make decisions about where to feed and how long to stay in the CCS as either a direct result of the environment or based on changes in body condition due to feeding.

## Conclusion

Albacore tuna are opportunistic predators that can successfully consume a variety of prey items. The C:N content of albacore white muscle tissue, which reflects fat stores and energy reserves for use in growth and migration, increased with albacore fork length and month. If time and size are accounted for, seasonal environmental conditions within the northern CCS also impacted C:N ratios, which decreased with SST and increased with upwelling intensity. The mean energy density of prey was not an important explanatory variable, but future research may clarify the relationship between prey composition and predator body condition. Tracking albacore body condition allows us to assess foraging success, which in turn indicates how well provisioned albacore are for their annual migrations and whether the CCS remains a sufficient foraging ground for their needs into the future.

## Supporting information

S1 Table Regression equations used to obtain prey lengths, masses, and energetic values from prey measurements. The equation and definition of all variables are given for each regression. The references (“ref”) are also provided.
(DOCX)

S2 Table Summary of albacore white muscle tissue samples for C:N and AFDW analysis by year. (DOCX)

S3 Table Summary of albacore stomachs sampled and fork length (FL) by year. (DOCX)

S1 Fig Pearson’s *r* correlation matrix of potential predictors of individual albacore muscle tissue C:N.included in the GAM. Significant coefficients (p < 0.05) are marked with an *.
(TIF)

S2 Fig Scatter plots of relative condition, AFDW, and C:N by year. Scatter plots of CCS albacore tuna measured mass divided by expected mass (relative condition), percent ash free dry weight (AFDW), and carbon to nitrogen mass ratio (C:N) by year.
(TIF)

S1 Data Spreadsheet containing underlying data. Column names are defined on the first sheet.
(XLSX)

## References

[1] Alerstam T, Hedenström A, Åkesson S. Long‐distance migration: evolution and determinants. Oikos. 2003;103(2):247–60. doi: 10.1034/j.1600-0706.2003.12559.x

[2] Anderson JJ, Gurarie E, Bracis C, Burke BJ, Laidre KL. Modeling climate change impacts on phenology and population dynamics of migratory marine species. Ecological Modelling. 2013;264:83–97. doi: 10.1016/j.ecolmodel.2013.03.009

[3] Gleiber MR, Hardy NA, Morganson CJ, Nickels CF, Muhling BA, Portner EJ, et al. Trait-based indicators of resource selection by albacore tuna in the California Current Large Marine Ecosystem. Ecological Indicators. 2024;158:111473. doi: 10.1016/j.ecolind.2023.111473

[4] Gleiber MR, Hardy NA, Roote Z, Krug-MacLeod AM, Morganson CJ, Tandy Z, et al. The Pelagic Species Trait Database, an open data resource to support trait-based ocean research. Sci Data. 2024;11(1):2. doi: 10.1038/s41597-023-02689-9 38216562 PMC10786825

[5] Hallier J, Gaertner D. Drifting fish aggregation devices could act as an ecological trap for tropical tuna species. Mar Ecol Prog Ser. 2008;353:255–64. doi: 10.3354/meps07180

[6] Golet W, Record N, Lehuta S, Lutcavage M, Galuardi B, Cooper A, et al. The paradox of the pelagics: why bluefin tuna can go hungry in a sea of plenty. Mar Ecol Prog Ser. 2015;527:181–92. doi: 10.3354/meps11260

[7] Snyder S, Franks PJS, Talley LD, Xu Y, Kohin S. Crossing the line: Tunas actively exploit submesoscale fronts to enhance foraging success. Limnol Oceanogr Letters. 2017;2(5):187–94. doi: 10.1002/lol2.10049

[8] Sinclair EH, Pelland NA, Johnson DS. Community composition and spatial energetics of mesopelagic fishes and squids in the eastern Bering Sea as influenced by habitat variables. Deep Sea Research Part I: Oceanographic Research Papers. 2022;182:103704. doi: 10.1016/j.dsr.2022.103704

[9] Preti A, Stohs SM, DiNardo GT, Saavedra C, MacKenzie K, Noble LR, et al. Feeding ecology of broadbill swordfish (Xiphias gladius) in the California current. PLoS One. 2023;18(2):e0258011. doi: 10.1371/journal.pone.0258011 36795680 PMC9934375

[10] Block BA, Jonsen ID, Jorgensen SJ, Winship AJ, Shaffer SA, Bograd SJ, et al. Tracking apex marine predator movements in a dynamic ocean. Nature. 2011;475(7354):86–90. doi: 10.1038/nature10082 21697831

[11] Brodeur R, Hunsicker M, Hann A, Miller T. Effects of warming ocean conditions on feeding ecology of small pelagic fishes in a coastal upwelling ecosystem: a shift to gelatinous food sources. Mar Ecol Prog Ser. 2019;617–618:149–63. doi: 10.3354/meps12497

[12] Daly EA, Brodeur RD. Warming Ocean Conditions Relate to Increased Trophic Requirements of Threatened and Endangered Salmon. PLoS One. 2015;10(12):e0144066. doi: 10.1371/journal.pone.0144066 26675673 PMC4682959

[13] McClatchie S, Field J, Thompson AR, Gerrodette T, Lowry M, Fiedler PC, et al. Food limitation of sea lion pups and the decline of forage off central and southern California. R Soc Open Sci. 2016;3(3):150628. doi: 10.1098/rsos.150628 27069651 PMC4821262

[14] Piatt JF, Parrish JK, Renner HM, Schoen SK, Jones TT, Arimitsu ML, et al. Extreme mortality and reproductive failure of common murres resulting from the northeast Pacific marine heatwave of 2014-2016. PLoS One. 2020;15(1):e0226087. doi: 10.1371/journal.pone.0226087 31940310 PMC6961838

[15] Childers J, Snyder S, Kohin S. Migration and behavior of juvenile North Pacific albacore (Thunnus alalunga). Fisheries Oceanography. 2011;20(3):157–73. doi: 10.1111/j.1365-2419.2011.00575.x

[16] Muhling BA, Snyder S, Hazen EL, Whitlock RE, Dewar H, Park JY, et al. Risk and Reward in Foraging Migrations of North Pacific Albacore Determined From Estimates of Energy Intake and Movement Costs. Front Mar Sci. 2022;9. doi: 10.3389/fmars.2022.730428

[17] Nickels CF, Portner EJ, Snodgrass O, Muhling B, Dewar H. Juvenile Albacore tuna (Thunnus alalunga) foraging ecology varies with environmental conditions in the California Current Large Marine Ecosystem. Fisheries Oceanography. 2023;32(5):431–47. doi: 10.1111/fog.12638

[18] Frawley TH, Muhling BA, Brodie S, Fisher MC, Tommasi D, Le Fol G, et al. Changes to the structure and function of an albacore fishery reveal shifting social‐ecological realities for Pacific Northwest fishermen. Fish and Fisheries. 2020;22(2):280–97. doi: 10.1111/faf.12519

[19] Rasmussen RS, Morrissey MT, Carroll S. Effect of Seasonality, Location, and Size on Lipid Content in North Pacific Troll-Caught Albacore Tuna (Thunnus alalunga). J Aquatic Food Product Technol. 2006;15(2):73–86. doi: 10.1300/j030v15n02_07

[20] Goñi N, Arrizabalaga H. Seasonal and interannual variability of fat content of juvenile albacore (Thunnus alalunga) and bluefin (Thunnus thynnus) tunas during their feeding migration to the Bay of Biscay. Progress Oceanography. 2010;86(1–2):115–23. doi: 10.1016/j.pocean.2010.04.016

[21] Post DM, Layman CA, Arrington DA, Takimoto G, Quattrochi J, Montaña CG. Getting to the fat of the matter: models, methods and assumptions for dealing with lipids in stable isotope analyses. Oecologia. 2007;152(1):179–89. doi: 10.1007/s00442-006-0630-x 17225157

[22] Weil J, Trudel M, Tucker S, Brodeur RD, Juanes F. Percent ash-free dry weight as a robust method to estimate energy density across taxa. Ecol Evol. 2019;9(23):13244–54. doi: 10.1002/ece3.5775 31871642 PMC6912885

[23] Cren EDL. The Length-Weight Relationship and Seasonal Cycle in Gonad Weight and Condition in the Perch (Perca fluviatilis). J Animal Ecol. 1951;20(2):201. doi: 10.2307/1540

[24] Ricker WE. Linear Regressions in Fishery Research. J Fish Res Bd Can. 1973;30(3):409–34. doi: 10.1139/f73-072

[25] Ricker WE. Computation and interpretation of biological statistics of fish populations. Fish Res Board of Can Bull. 1975;191:1–382.

[26] Logan JM, Jardine TD, Miller TJ, Bunn SE, Cunjak RA, Lutcavage ME. Lipid corrections in carbon and nitrogen stable isotope analyses: comparison of chemical extraction and modelling methods. J Anim Ecol. 2008;77(4):838–46. doi: 10.1111/j.1365-2656.2008.01394.x 18489570

[27] Marshall MR. Ash analysis. In: Nielsen S, editor. Food analysis. 4th ed. New York: Springer. 2010. p. 105–16.

[28] Crane DP, Killourhy CC, Clapsadl MD. Effects of three frozen storage methods on wet weight of fish. Fisheries Research. 2016;175:142–7. doi: 10.1016/j.fishres.2015.11.022

[29] Clarke MR. A handbook for the identification of cephalopod beaks. Clarke MR, editor. Oxford: Clarendon Press. 1986.

[30] Clothier CR. A key to some southern California fishes based on vertebral characters. California Division of Fish and Game, Fish Bull. 1950;79:1–83.

[31] Harvey JT, Loughlin TR, Perez MA, Oxman DS. Relationship between fish size and otolith length for 63 species of fishes from the Eastern North Pacific Ocean. NOAA Technical Report NMFS. 2000;150 https://repository.library.noaa.gov/view/noaa/3159/noaa_3159_DS1.pdf

[32] Isaacs J, Fleminger A, Miller J. Distributional atlas of zooplankton biomass in the California Current region: Spring and fall 1955–1959. 1969. https://www.calcofi.org/publications/atlases/CalCOFI_Atlas_10.pdf

[33] Lowry MS. Photographic catalog of California marine fish otoliths: Prey of California sea lions (Zalophus californianus). NOAA-TM-NMFS-SWFSC-483. NOAA. 2011. https://repository.library.noaa.gov/view/noaa/4511/noaa_4511_DS1.pdf

[34] Pinkas L, Oliphant MS, Iverson ILK. Food habits of albacore, bluefin tuna, and bonito in California waters. Fish Bull. 1971;152(10):1–105.

[35] Wolff GA. Identification and estimation of size from the beaks of 18 species of cephalopods from the Pacific Ocean. 1984.17. https://repository.library.noaa.gov/view/noaa/5605/noaa_5605_DS1.pdf

[36] Glaser SM, Waechter KE, Bransome NC. Through the stomach of a predator: Regional patterns of forage in the diet of albacore tuna in the California Current System and metrics needed for ecosystem-based management. J Marine Syst. 2015;146:38–49. doi: 10.1016/j.jmarsys.2014.07.019

[37] Portner EJ, Snodgrass O, Dewar H. Pacific bluefin tuna, Thunnus orientalis, exhibits a flexible feeding ecology in the Southern California Bight. PLoS One. 2022;17(8):e0272048. doi: 10.1371/journal.pone.0272048 36006923 PMC9409590

[38] R Core Team. R: A language and environment for statistical computing. 2024.

[39] Wood SN. Fast Stable Restricted Maximum Likelihood and Marginal Likelihood Estimation of Semiparametric Generalized Linear Models. J Royal Statistical Society Series B: Statistical Methodol. 2010;73(1):3–36. doi: 10.1111/j.1467-9868.2010.00749.x

[40] Wood SN. On confidence intervals for generalized additive models based on penalized regression splines. Aus NZ J of Statistics. 2006;48(4):445–64. doi: 10.1111/j.1467-842x.2006.00450.x

[41] Jacox MG, Edwards CA, Hazen EL, Bograd SJ. Coastal Upwelling Revisited: Ekman, Bakun, and Improved Upwelling Indices for the U.S. West Coast. JGR Oceans. 2018;123(10):7332–50. doi: 10.1029/2018jc014187

[42] Muhling BA, Brodie S, Snodgrass O, Tommasi D, Jacox M. Dynamic habitat use of albacore and their primary prey species in the California current system. CalCOFI Reports. 2019;60:79–93.

[43] Ainley DG, Sydeman WJ, Parrish RH, Lenarz WH. Oceanic factors influencing distribution of young rockfish (Sebastes) in central California: A predator’s perspective. CalCOFI Reports. 1993;34:133–9.

[44] Cimino MA, Santora JA, Schroeder I, Sydeman W, Jacox MG, Hazen EL, et al. Essential krill species habitat resolved by seasonal upwelling and ocean circulation models within the large marine ecosystem of the California Current System. Ecography. 2020;43(10):1536–49. doi: 10.1111/ecog.05204

[45] Mendelssohn R. RerddapXtracto: Extracts Environmental Data from “ERDDAP™” Web Services. CRAN: Contributed Packages. The R Foundation. 2019. doi: 10.32614/cran.package.rerddapxtracto

[46] JPL MUR MEaSUREs Project. GHRSST level 4 MUR global foundation sea surface temperature analysis. CA, USA: PO.DAAC. 2015. doi: 10.5067/GHGMR-4FJ04

[47] Hu C, Lee Z, Franz B. Chlorophyll-a algorithms for oligotrophic oceans: A novel approach based on three‐band reflectance difference. J Geophys Res. 2012;117(C1). doi: 10.1029/2011jc007395

[48] Marra G, Wood SN. Practical variable selection for generalized additive models. Computational Statist Data Analysis. 2011;55(7):2372–87. doi: 10.1016/j.csda.2011.02.004

[49] Oliver ECJ, Donat MG, Burrows MT, Moore PJ, Smale DA, Alexander LV, et al. Longer and more frequent marine heatwaves over the past century. Nat Commun. 2018;9(1):1324. doi: 10.1038/s41467-018-03732-9 29636482 PMC5893591

[50] Welch H, Savoca MS, Brodie S, Jacox MG, Muhling BA, Clay TA, et al. Impacts of marine heatwaves on top predator distributions are variable but predictable. Nat Commun. 2023;14(1):5188. doi: 10.1038/s41467-023-40849-y 37669922 PMC10480173

[51] Thompson AR, Swalethorp R, Alksne M, Santora JA, Hazen EL, Leising A, et al. State of the California Current Ecosystem report in 2022: a tale of two La Niñas. Front Mar Sci. 2024;11. doi: 10.3389/fmars.2024.1294011

[52] Blank JM, Morrissette JM, Farwell CJ, Price M, Schallert RJ, Block BA. Temperature effects on metabolic rate of juvenile Pacific bluefin tuna Thunnus orientalis. J Exp Biol. 2007;210(Pt 23):4254–61. doi: 10.1242/jeb.005835 18025023

[53] Whitlock RE, Hazen EL, Walli A, Farwell C, Bograd SJ, Foley DG, et al. Direct quantification of energy intake in an apex marine predator suggests physiology is a key driver of migrations. Sci Adv. 2015;1(8):e1400270. doi: 10.1126/sciadv.1400270 26601248 PMC4643779

[54] Lluch-Belda DA, Lluch-Cota DB, Hernandez-Vazquez SE, Salinas-Zavala CA, Schwartzlose RA. Sardine and anchovy spawning as related to temperature and upwell in the California current system. CalCOFI Reports. 1991;32:105–11.

[55] Véron M, Duhamel E, Bertignac M, Pawlowski L, Huret M. Major changes in sardine growth and body condition in the Bay of Biscay between 2003 and 2016: Temporal trends and drivers. Progress Oceanography. 2020;182:102274. doi: 10.1016/j.pocean.2020.102274

[56] Jacox MG, Alexander MA, Stock CA, Hervieux G. On the skill of seasonal sea surface temperature forecasts in the California Current System and its connection to ENSO variability. Clim Dyn. 2017;53(12):7519–33. doi: 10.1007/s00382-017-3608-y

[57] Deser C, Alexander MA, Xie S-P, Phillips AS. Sea surface temperature variability: patterns and mechanisms. Ann Rev Mar Sci. 2010;2:115–43. doi: 10.1146/annurev-marine-120408-151453 21141660

[58] Venegas RM, Strub PT, Beier E, Letelier R, Thomas AC, Cowles T, et al. Satellite‐derived variability in chlorophyll, wind stress, sea surface height, and temperature in the northern California current system. J Geophys Res. 2008;113(C3). doi: 10.1029/2007jc004481

[59] Gentemann CL, Fewings MR, García‐Reyes M. Satellite sea surface temperatures along the West Coast of the United States during the 2014–2016 northeast Pacific marine heat wave. Geophysical Res Lett. 2017;44(1):312–9. doi: 10.1002/2016gl071039

[60] Ménard F, Marchal E. Foraging behaviour of tuna feeding on small schooling Vinciguerria nimbaria in the surface layer of the equatorial Atlantic Ocean. Aquatic Living Res. 2003;16(3):231–8. doi: 10.1016/s0990-7440(03)00040-8

[61] Brett JR. Energetics. In: Groot C, Margolis L, Clarke WC, editors. Physiological ecology of Pacific Salmon. Vancouver: University of British Columbia Press. 1995.

[62] Craig JF. The Body Composition of Adult Perch, Perca fluviatilis in Windermere, with Reference to Seasonal Changes and Reproduction. J Animal Ecol. 1977;46(2):617. doi: 10.2307/3834

[63] Dotson RC. Fat deposition and utilization in albacore. Physiological Ecol Tunas. Elsevier. 1978. p. 343–55. doi: 10.1016/b978-0-12-639180-0.50029-x

[64] Griffiths SP, Fry GC, Manson FJ, Pillans RD. Feeding dynamics, consumption rates and daily ration of longtail tuna (Thunnus tonggol) in Australian waters, with emphasis on the consumption of commercially important prawns. Mar Freshwater Res. 2007;58(4):376. doi: 10.1071/mf06197

[65] Griffiths SP, Kuhnert PM, Fry GF, Manson FJ. Temporal and size-related variation in the diet, consumption rate, and daily ration of mackerel tuna (Euthynnus affinis) in neritic waters of eastern Australia. ICES J Marine Sci. 2009;66(4):720–33. doi: 10.1093/icesjms/fsp065

[66] Butler CM, Rudershausen PJ, Buckel JA. Feeding ecology of Atlantic bluefin tuna (Thunnus thynnus) in North Carolina: diet, daily ration, and consumption of Atlantic menhaden (Brevoortia tyrannus). Fish Bull. 2010;108(1):56–69.

[67] Clark TD, Brandt WT, Nogueira J, Rodriguez LE, Price M, Farwell CJ, et al. Postprandial metabolism of Pacific bluefin tuna (Thunnus orientalis). J Exp Biol. 2010;213(Pt 14):2379–85. doi: 10.1242/jeb.043455 20581267

[68] Madigan DJ, Litvin SY, Popp BN, Carlisle AB, Farwell CJ, Block BA. Tissue turnover rates and isotopic trophic discrimination factors in the endothermic teleost, pacific bluefin tuna (Thunnus orientalis). PLoS One. 2012;7(11):e49220. doi: 10.1371/journal.pone.0049220 23145128 PMC3492276

[69] Varela JL, Cañavate JP, Medina A, Mourente G. Inter-regional variation in feeding patterns of skipjack tuna (Katsuwonus pelamis) inferred from stomach content, stable isotope and fatty acid analyses. Marine Environ Res. 2019;152:104821. doi: 10.1016/j.marenvres.2019.104821 31653436

[70] Pozo Buil M, Jacox MG, Fiechter J, Alexander MA, Bograd SJ, Curchitser EN, et al. A dynamically downscaled ensemble of future projections for the California current system. Front Mar Sci. 2021;8. doi: 10.3389/fmars.2021.612874

[71] Jacox MG, Bograd SJ, Fiechter J, Pozo Buil M, Alexander M, Amaya D, et al. Linking upwelling dynamics and subsurface nutrients to projected productivity changes in the California current system. Geophysical Research Lett. 2024;51(10). doi: 10.1029/2023gl108096

[72] Hardy NA, Matuch C, Roote Z, George I, Muhling BA, Jacox MG, et al. Trait‐based analyses reveal global patterns in diverse diets of albacore tuna (*Thunnus alalunga*). Fish Fisheries. 2023;25(2):268–82. doi: 10.1111/faf.12807

[73] Guiry EJ, Szpak P. Quality control for modern bone collagen stable carbon and nitrogen isotope measurements. Methods Ecol Evol. 2020;11(9):1049–60. doi: 10.1111/2041-210x.13433

