# Supplementary material for: Ocean conditions drive interannual variability in juvenile albacore tuna (Thunnus alalunga) muscle energy content in the California Current System
Source: PLoS One. 2025 Sep 11;20(9):e0331436. doi: 10.1371/journal.pone.0331436 (PMC12425301; doi:10.1371/journal.pone.0331436)
Supplement: S1 Table — The equation and definition of all variables are given for each regression. The references (“ref”) are also provided. (DOCX) [file pone.0331436.s001.docx]

Table S1: Regressions used to obtain prey lengths, masses, and energetic values from prey measurements. The equation and definition of all variables are given for each regression. The references (“ref”) are also provided.

|  |  | **Length** | | | **Weight** | | | | **Energy density (kJ/g wet weight)** | |
| --- | --- | --- | --- | --- | --- | --- | --- | --- | --- | --- |
|  |  | equation | *a* | ref | equation | *a* | *b* | ref | *a* | ref |
| **Family** | **Prey ID** | y = a*x |  |  | y = a*x^b |  |  |  |  |  |
|  |  |  |  |  |  |  |  |  |  |  |
| ***Fishes*** |  |  |  |  |  |  |  |  |  |  |
| Carangidae | *Trachurus symmetricus* | x = VCL (cm)  y = SL (cm) | 1.1 | Glaser 2010 | x = SL (cm)  y = BW (g) | 0.0635 | 2.556 | Harvey et al 2000 | 6.4 | Gleiber et al 2022 |
| Centrolophidae | *Icichthys lockingtoni* | x = VCL (cm)  y = SL (cm) | 1.1 | Glaser 2010 | x = SL (cm)  y = BW (g) | 0.024725 | 3.025 | Family median, fishbase | 5.76* | see unidentified |
| Clupeidae | *Sardinops sagax* | x = VCL (cm)  y = SL (cm) | 1.3 | Glaser 2010 | x = SL (cm)  y = BW (g) | 0.00763 | 3.15 | fishbase | 5.3 | Gleiber et al 2022 |
| Engraulidae | *Engraulis mordax* | x = VCL (cm)  y = SL (cm) | 1.1 | Glaser 2010 | x = SL (cm)  y = BW (g) | 0.0485 | 2.413 | Harvey et al 2000 | 5.58 | Gleiber et al 2022 |
| Microstomatidae | *Nansenia* sp. | x = VCL (cm)  y = SL (cm) | 1.1 | Glaser 2010 | x = SL (mm)**  y = BW (g) | 0.000033 | 2.729 | **Nansenia oblita* Battaglia et al 2010 | 10.4 | **Nansenia candida* from Sinclair et al 2015 |
| Myctophidae | *Tarletonbeania crenularis* | x = VCL (cm)  y = SL (cm) | 1.1 | Glaser 2010 | x = SL (cm)  y = BW (g) | 0.007155 | 3.11865 | Family median, fishbase | 4.6 | Gleiber et al 2022 |
| Ophidiidae | *Chilara taylori* | x = VCL (cm)  y = SL (cm) | 1.1 | Glaser 2010 | x = SL (cm)  y = BW (g) | 0.0004 | 3.761 | Harvey et al 2000 | 3.39 | Spear 1993 |
| Paralepididae | *Lestidiops ringens* | x = VCL (cm)  y = SL (cm) | 1.1 | Glaser 2010 | x = SL (cm)  y = BW (g) | 0.00128 | 2.928 | Family median, fishbase | 5.76* | see unidentified |
| Pleuronectiformes | *Citharichthys sordidus* | x = VCL (cm)  y = SL (cm) | 1.1 | Glaser 2010 | x = SL (cm)  y = BW (g) | 0.00112 | 3.25 | Family median, fishbase | 3.32 | Gleiber et al 2022 |
| Scomberesocidae | *Cololabis saira* | x = VCL (cm)  y = SL (cm) | 1.2 | Glaser 2010 | x = KL* (mm) **  y = BW (g) | 0.000001653 | 3.172 | Median from Hughes 1974 | 7.02 | Gleiber et al 2022 |
| Scombridae | *Scomber japonicus* | x = VCL (cm)  y = SL (cm) | 1.2 | Glaser 2010 | x = SL (cm)  y = BW (g) | 0.10 | 3.10 | Furuichi et al, 2021 | 6.67 | Gleiber et al 2022 |
| Sebastidae | *Sebastes* spp. | x = VCL (cm)  y = SL (cm) | 1.1 | Glaser 2010 | x = SL (cm)  y = BW (g) | 0.0287 | 2.985 | Family median, fishbase | 4.88 | Gleiber et al 2022 |
|  | Unidentified fishes | x = VCL (cm)  y = SL (cm) | 1.1 | Glaser 2010 | x = SL (cm)  y = BW (g) | 0.0247 | 3.06 | Median of all regressions used of form y = a*x^b | 5.76 | Mean of all unique energy density values referenced here (n=10) |

- * KL = knob length. Used SL as KL was not available for our specimens.
- Family median from fishbase
  - Subset for family of interest and regressions built using Standard Lengths (SL) for which values of *a* and *b* are present.
- Median of all regressions used of form y = a*x^b
  - Using all regressions (except *Cololabis* from Hughes because KL in mm), but only one for each family so as not to skew median calculation towards fish identified with relatively high taxonomic resolution (only used single entry of *a* and *b* for Myctophidae and Plueronecti(dae/formes))

Table S1 continued: For prey for which regressions could not be found, the identity of an appropriate analog that was used is indicated with an asterisk.

|  |  | **Length (mm)** | | | | **Weight (g)** | | | | **Energy density (kJ)** | | |
| --- | --- | --- | --- | --- | --- | --- | --- | --- | --- | --- | --- | --- |
|  |  | type | *a* | *b* | ref | type | *a* | *b* | ref | | *a* | ref |
| **Family** | **Prey ID** |  |  |  |  |  |  |  |  | |  |  |
|  |  |  |  |  |  |  |  |  |  | |  |  |
| ***Cephalopods*** | |  |  |  |  |  |  |  |  | |  |  |
| Amphitretidae | *Japetella heathi* | x = BW(g)  y=ML (mm)  y = (x/a)^b | 9.97 E^-05^ | 0.3355705 | Schwarz et al 2020 | UHL (mm) to BW(g)  y = (x/a)^b | 22.08 | 0.7042254 | Schwarz et al 2020 | | 0.32 | Chen et al 2022 |
| Argonautidae | *Argonauta* sp.  **Ocythoe tuberculata* | UHL(mm) to ML(mm)  y = a*x+b | 4.47 | 0.83 | Lu and Ickeringill 2002 | UHL (mm) to BW (g)  y = x^a/(e^b) | 2.67 | 2.14 | Lu and Ickeringill 2002 | | 4.93 | Chen et al 2022 |
| Octopodidae | *Octopus bimaculatus*  **Octopus kaurna* | UHL (mm) to ML (mm)  y = a*x+b | 18.54 | 0.72 | Lu and Ickeringill 2002 | UHL (mm) to BW (g)  y = x^a*e^b | 2.77 | 1.14 | Lu and Ickeringill 2002 | | 3.35 | **Octopus rubescens* from  Anderson 2016 |
|  | *Octopus rubescens*  ** Octopus kaurna* | UHL (mm) to ML (mm)  y = a*x+b | 18.54 | 0.72 | Lu and Ickeringill 2002 | UHL (mm) to BW (g)  y = x^a*e^b | 2.77 | 1.14 | Lu and Ickeringill 2002 | | 3.35 | **Octopus rubescens*. from  Anderson 2016 |
| Octopoda | Unidentified octopus | UHL (mm) to ML (mm)  y = a*x+b | 11.51 | 0.78 | Median Argonautidae and Octopodidae regressions used of form  y = a*x+b | UHL (mm) to BW (g)  y = x^a*e^b | Need to use median of values that come out of regressions because equations are variable | | | | 2.87 | Mean of all unique energy density values referenced here (n=3) |
| Enoplotethidae | *Abraliopsis affinis* | LRL (mm*) to ML (mm)  y = a*x+b | 19.28 | 9.8 | Wolff 1984 | LRL (cm) to BW (g)  y = x^a*e^b | 2.1 | 5.5 | Wolff 1984 | | 4.02 | Chen et al 2022 |
|  | *Abraliopsis felis* | LRL (mm*) to ML (mm)  y = a*x+b | 40.55 | -2.66 | Wolff 1984 | LRL (cm) to BW (g)  y = x^a*e^b | 2.49 | 6.58 | Wolff 1984 | | 4.02 | Chen et al 2022 |
| Gonatidae | *Berryteuthis* sp.  **Berryteuthis magister* | LRL (mm) to ML (mm)  y = a*x+b | 43.99 | -5.14 | Clarke et al 1986 | LRL (mm) to BW (g)  y = x^a*e^b | 2.605 | 1.618 | Clarke et al 1986 | | 4.81 | Gleiber et al 2022 |
|  | *Gonatopsis* sp.  **Gonatopsis borealis* | LRL (mm) to ML (mm)  y = a*x+b | 38.14 | 2.11 | Sinclair et al 2015 | ML (mm) to BW (g)  Y = ax^b | 7.142 E^-05^ | 2.872 | Sinclair et al 2015 | | 4.10 | Gleiber et al 2022 |
|  | *Gonatus* sp.  **Gonatus* *middendorfi* | LRL (mm) to ML (mm)  y = a*x+b | 47.51 | 1.72 | Sinclair et al 2015 | ML (mm) to BW (g)  Y = ax^b | 1.39 E^-04^ | 2.552 | Sinclair et al 2015 | | 4.9 | Gleiber et al 2022 |
|  | *Gonatus* sp.  **Gonatus* onyx | LRL (mm*) to ML (mm)  y= a*x+b | 19.02 | 12.82 | Wolff 1984 | LRL (cm) to BW (g)  y = x^a*e^b | 2.13 | 4.99 | Wolff 1984 | | 4.9 | Gleiber et al 2022 |
| Histioteuthidae | *Histioteuthis heteropsis* | LRL (mm*) to ML (mm)  y = a*x+b | 20.57 | 2.04 | Wolff 1984 | LRL (cm) to BW (g)  y = x^a*e^b | 2.64 | 7.43 | Wolff 1984 | | 1.92 | Gleiber et al 2022 |
| Loliginidae | *Doryteuthis opalescens* | LRL (mm) to ML (mm)  y = a*x+b | 60.78 | 32.4 | Wolff 1984* was Loligo | LRL (cm) to BW (g)  y = x^a*e^b | 1.4 | 6.0 | Wolff 1984* was Loligo | | 4.42 | Gleiber et al 2022 |
| Mastigoteuthidae | *Mastigoteuthis dentata*  **Mastigoteuthis* spp. | LRL (mm) to ML (mm)  y = a*x+b | 29.08 | -1.8 | Clarke et al 1986 | LRL (mm) to BW (g)  y = x^a*e^b | 2.88 | 0.184 | Clarke et al 1986 | | 1.91 | Gleiber et al 2022 |
| Octopoteuthidae | *Octopoteuthis* sp. | LRL (mm) to ML (mm)  y = a*x+b | 18.55 | -1.51 | Lu and Ickeringill 2002 | LRL (cm) to BW (g)  y = x^a*e^b | 2.54 | 0.23 | Lu and Ickeringill 2002 | | 3.08 | Clarke et al 1985 |
| Onychoteuthidae | *Onychoteuthis borealijaponica* | LRL (mm) to ML (mm)  y = a*x+b | 50.99 | -19.893 | Lowry et al 2020 | LRL (mm) to BW (g)  y = ax^b | 1.11255 E ^-04^ | 2.7555 | Median of male and female from Bigelow 1994 | | 4.92 | Gleiber et al 2022 |
| Decapodiformes | Unidentified squids | LRL (mm) to ML (mm) | 38.14 | 1.720 | Median of all squid regressions used of form  y = a*x+b | LRL (cm) to BW (g) | Need to use median of values that come out of regressions because units of x and equations are variable | | | | 3.79 | Mean of all unique squid energy density values referenced here (n=9) |
|  |  |  |  |  |  |  |  |  |  | |  |  |
| ***Crustaceans*** | |  |  |  |  |  |  |  |  | |  |  |
| Euphausiidae | Euphausiidae  *Euphausia pacifica*  *Nematoscelis difficilis*  *Thysanoessa spinifera* | TL measured | - | - | - | constant | - | 0.08 | * median value for  Euphausiid of approximately 20mm TL  Kulka and Corey 1982 | | 3.09 | * *Euphausia pacifica* from Gleiber et al 2022 |
| Hyperiidae | Amphipoda  Hyperiidea  *Phronima* sp.  Oxycephalidae | TL measured | - | - | - | constant | - | 0.1 | * median value for  Phronima from Bishop and Geiger 2006 | | 2.4 | * *Hyperiidae* spp. From Gleiber et al 2022 |
| Munididae | Munididae  **Pleuroncodes planipes* | y = TL (mm)  x = CL (mm)  y = x*a | 2.45 | - | Boyd 1962 | CL (mm) to BW(g)  Y = ax^b | 0.00095 | 2.755 | Median from Rodriguez-Jaramillo et al 2018 | | 5.64 | Gleiber et al 2022 |
| Malacostraca | Malacostraca  Decapoda  Pleocyemata  Sergestidae | TL measured | - | - | - | constant | - | 0.1 | Glaser 2010 | | 3.2 | *Crustaceans (other) from Glaser 2010 |
| Gastropoda | Pteropoda  Gastropoda  Pterotracheoidea | - | - | - | - | constant | - | 2 | Glaser 2010 | | 1.84 | **Limacin*a spp. from Gleiber et al 2022 |
| Thaliacea | Thaliacea  Salpidae | - | - | - | - | constant | - | 2 | Glaser 2010 | | 0.55 | **Pegea* spp. From Gleiber et al 2022 |
|  | Bivalvia | - | - | - | - | constant | - | 2 | Glaser 2010 | | 0.55 | **Pegea* spp. From Gleiber et al 2022 |
|  | Siphonophorae | - | - | - | - | constant | - | 2 | Glaser 2010 | | 0.55 | **Pegea* spp. From Gleiber et al 2022 |

## References

Anderson ES. The Response of a Predatory Fish, Ophiodon elongatus, to a Marine Protected Area: Variation in Diet, Catch Rates, and Size Composition. California Polytechnic State University 2016; 32. doi:10.15368/theses.2016.153

Bigelow KA. Age and growth of the oceanic squid Onychoteuthis borealijaponica in the North Pacific. Fish. Bull. 1994; 92:13-25.

Bishop RE, Geiger SP. Phronima Energetics: Is There a Bonus to the Barrel?. Crustaceana. 2006 Oct 1: 79(9):1059–1070.

Boettiger C, Lang DT, Wainwright PC. rfishbase: exploring, manipulating and visualizing FishBase data from R. J. Fish Biol. 2012 Nov; 81(6):2030-2039. doi: 10.1111/j.1095-8649.2012.03464.x

Boyd CM. The Biology of a Marine Decapod Crustacean, Pleuroncodes planipes Stimpson, 1860. UC San Diego: Scripps Institution of Oceanography*.* 1962. https://escholarship.org/content/qt80n8w92r/qt80n8w92r.pdf

Chen RS, Portner EJ, Choy CA. Gelatinous cephalopods as important prey for a deep-sea fish predator. Mar. Biol. 2022 Nov 16; 169(12):155 doi:10.1007/s00227-022-04116-w

Clarke A, Clarke MR, Holmes LJ, Waters TD. Calorific Values and Elemental Analysis of Eleven Species of Oceanic Squids (Mollusca:Cephalopoda). J. Mar. Biol. Assoc. U. K. 1985 Nov; 65(4):983-986. doi:10.1017/S0025315400019457

Furuichi S, Kamimura Y, Yukami R. Length–length and Length–weight Relationships for Four Dominant Small Pelagic Fishes in the Kuroshio–Oyashio Current System. Thalassas. 2021 Oct; 37:651–657. doi:10.1007/s41208-021-00300-9

Glaser SM. Interdecadal variability in predator–prey interactions of juvenile North Pacific albacore in the California Current System. Mar Ecol Prog Ser. 2010 Sep 13; 414:209-221. doi: 10.3354/meps08723

Gleiber MR, Hardy NA, Roote Z, Krug-MacLeod AM, Morganson CJ, Tandy Z, et al. (2024b). The Pelagic Species Trait Database, an open data resource to support trait-based ocean research. Sci. Data. 2024 Jan 12; 11(1): 2. doi: 10.1038/s41597-023-02689-9

Harvey JT, Loughlin TR., Perez MA, Oxman DS. Relationship between fish size and otolith length for 63 species of fishes from the Eastern North Pacific Ocean. NOAA Technical Report NMFS. 2000 Aug; 150. https://repository.library.noaa.gov/view/noaa/3159/noaa_3159_DS1.pdf

Hughes SE. 1974. Stock composition, growth, mortality, and availability of Pacific saury, Cololabis saira, of the northeastern Pacific Ocean. Fish. Bull. 1974; 22(1):121-131.

Kulka DW, Corey S. Length and Weight Relationships of Euphausiids and Caloric Values of Meganyctiphanes Norvegica (M. Sars) in the Bay of Fundy, J. Crustac. Biol. 1982 Apr 1; 2(2):239-247. doi:10.2307/1548004

Lu CC, Ickeringill R. Cephalopod beak identification and biomass estimation techniques: tools for dietary studies of southern Australian finfishes. Mus. Vic. Sci. Rep.2002; 6:1–65. doi:10.24199/j.mvsr.2002.06

Lowry MS, Curtis KA, Boerger CM. Measurements and regressions of otoliths, cephalopod beaks, and other prey hard parts used to reconstruct California Current predator diet composition. NOAA Technical Memo. 2020 Dec; NMFS-SWFSC-637. https://repository.library.noaa.gov/view/noaa/27914

Schwarz R, Piatkowski U, Robison BH, Laptikhovsky VV, Hoving HJ. Life history traits of the deep-sea pelagic cephalopods Japetella diaphana and Vampyroteuthis infernalis. Deep Sea Res., Part I. 2020 Oct 1; 164:103365. doi:10.1016/j.dsr.2020.103365

Sinclair EH, Walker WA, Thomason JR. Body Size Regression Formulae, Proximate Composition and Energy Density of Eastern Bering Sea Mesopelagic Fish and Squid. PLoS One. 2015 Aug 19; 10(8):e0132289. doi:10.1371/journal.pone.0132289

Spear LB. Dynamics and Effect of Western Gulls Feeding in a Colony of Guillemots and Brandt’s Cormorants. J. Anim. Ecol. 1993 Jul 1; 62(3):399–414. doi:10.2307/5190

Wolff GA. Identification and estimation of size from the beaks of 18 species of cephalopods from the Pacific Ocean. NOAA NMFS Technical Report. 1984 Nov; 17. https://repository.library.noaa.gov/view/noaa/5605/noaa_5605_DS1.pdf
