# Supplementary material for: Ocean conditions drive interannual variability in juvenile albacore tuna (Thunnus alalunga) muscle energy content in the California Current System
Source: PLoS One. 2025 Sep 11;20(9):e0331436. doi: 10.1371/journal.pone.0331436 (PMC12425301; doi:10.1371/journal.pone.0331436)
Supplement: S2 Table — (DOCX) [file pone.0331436.s002.docx]

Table S2: Summary of Albacore white muscle tissue samples for C:N and AFDW analysis by year.

| Year | *n* C:N | *n* realistic C:N | *n* realistic C:N with month | *n* realistic C:N with month, FL, and stomach (included in GAM) | *n* AFDW | *n* realistic C:N matched to AFDW |
| --- | --- | --- | --- | --- | --- | --- |
|  |  |  |  |  |  |  |
| **2012** | 20 | 19 | 9 | 9 |  |  |
| **2013** | 20 | 20 | 5 | 5 |  |  |
| **2014** | 20 | 19 | 9 | 6 | 26 | 9 |
| **2015** | 20 | 20 | 19 | 2 |  |  |
| **2017** | 20 | 20 | 12 | 11 |  |  |
| **2018** | 17 | 17 | 17 | 17 |  |  |
| **2019** | 20 | 20 | 13 | 13 |  |  |
| **2020** | 20 | 20 | 11 | 11 |  |  |
| **2021** | 20 | 20 | 20 | 19 | 29 | 15 |
| **2022** | 20 | 20 | 20 | 20 | 28 | 20 |
|  |  |  |  |  |  |  |
| **Total** | **197** | **195** | **135** | **113** | **83** | **44** |
