# Supplementary material for: Ocean conditions drive interannual variability in juvenile albacore tuna (Thunnus alalunga) muscle energy content in the California Current System
Source: PLoS One. 2025 Sep 11;20(9):e0331436. doi: 10.1371/journal.pone.0331436 (PMC12425301; doi:10.1371/journal.pone.0331436)
Supplement: S3 Table — (DOCX) [file pone.0331436.s003.docx]

Table S3: Summary of Albacore stomachs sampled and fork length (FL) by year.

| Year | *n* stomachs | *n* stomachs with prey | *n* stomachs with prey and realistic %W | FL range (cm) | FL mean ± SD (cm) |
| --- | --- | --- | --- | --- | --- |
|  |  |  |  |  |  |
| **2009** | 14 | 13 | 13 | 58-90 | 71 (±12) |
| **2010** | 121 | 85 | 82 | 54-83 | 65 (±5) |
| **2011** | 50 | 44 | 41 | 51-85 | 65 (±8) |
| **2012** | 50 | 36 | 35 | 54-80 | 65 (±7) |
| **2013** | 65 | 52 | 51 | 56-90 | 69 (±9) |
| **2014** | 38 | 37 | 36 | 51-88 | 66 (±12) |
| **2015** | 30 | 30 | 29 | 50-58 | 54 (±3) |
| **2017** | 24 | 20 | 20 | 53-84 | 68 (±8) |
| **2018** | 49 | 48 | 48 | 56-94 | 66 (±8) |
| **2019** | 53 | 53 | 53 | 53-80 | 66 (±6) |
| **2020** | 32 | 32 | 32 | 51-77 | 63 (±9) |
| **2021** | 55 | 53 | 53 | 55-86 | 70 (±10) |
| **2022** | 31 | 31 | 31 | 53-84 | 69 (±9) |
|  |  |  |  |  |  |
| **Total** | **612** | **534** | **524** | **50-94** | **66 (±8)** |
