# Supplementary figures and images for: Ocean conditions drive interannual variability in juvenile albacore tuna (Thunnus alalunga) muscle energy content in the California Current System
Source: PLoS One. 2025 Sep 11;20(9):e0331436. doi: 10.1371/journal.pone.0331436 (PMC12425301; doi:10.1371/journal.pone.0331436)

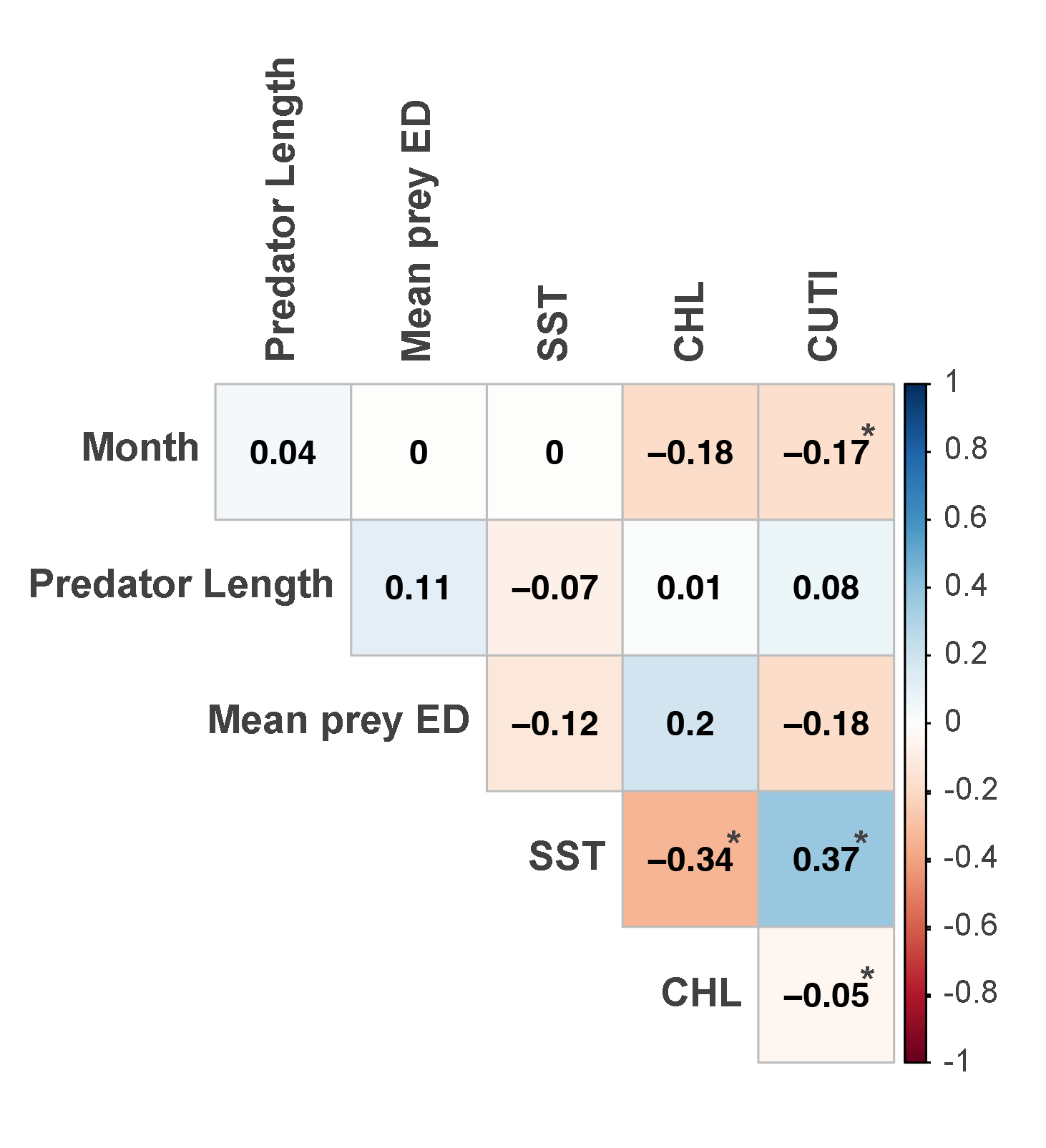

Supplement: S1 Fig — Significant coefficients (p < 0.05) are marked with an *. (TIF) [file pone.0331436.s004.tif]

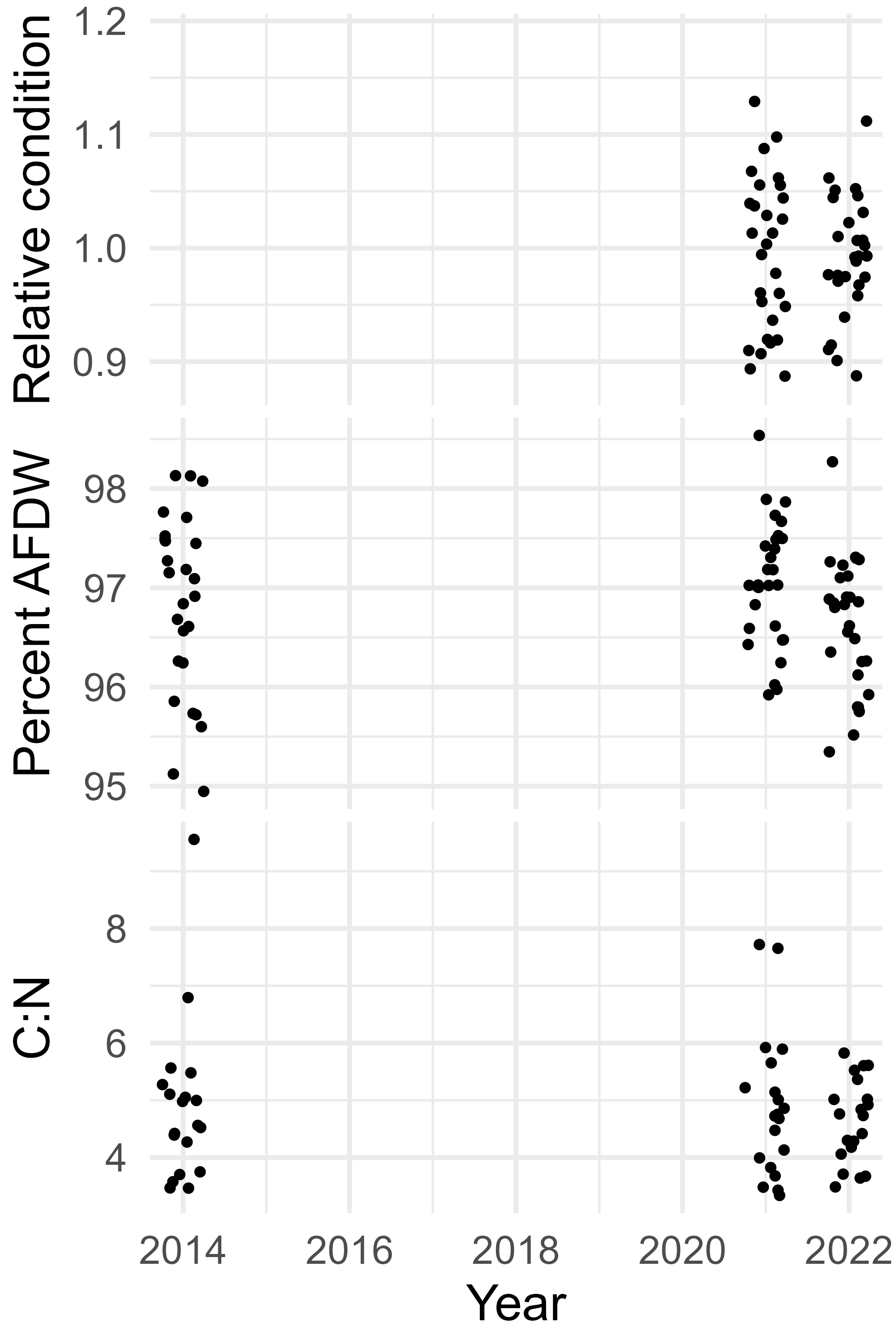

Supplement: S2 Fig — Scatter plots of CCS albacore tuna measured mass divided by expected mass (relative condition), percent ash free dry weight (AFDW), and carbon to nitrogen mass ratio (C:N) by year. (TIF) [file pone.0331436.s005.tif]
